# The Number and Distinct Clustering Patterns of Voltage-Gated Calcium Channels in Nerve Terminals

**DOI:** 10.3389/fnana.2022.846615

**Published:** 2022-02-24

**Authors:** Kohgaku Eguchi, Jacqueline Montanaro, Elodie Le Monnier, Ryuichi Shigemoto

**Affiliations:** Institute of Science and Technology Austria (IST Austria), Klosterneuburg, Austria

**Keywords:** presynaptic active zone, synaptic vesicle, electron microscopy, freeze-fracture replica labeling, clustering analysis, voltage-gated calcium channel

## Abstract

Upon the arrival of action potentials at nerve terminals, neurotransmitters are released from synaptic vesicles (SVs) by exocytosis. Ca_*V*_2.1, 2.2, and 2.3 are the major subunits of the voltage-gated calcium channel (VGCC) responsible for increasing intraterminal calcium levels and triggering SV exocytosis in the central nervous system (CNS) synapses. The two-dimensional analysis of Ca_*V*_2 distributions using sodium dodecyl sulfate (SDS)-digested freeze-fracture replica labeling (SDS-FRL) has revealed their numbers, densities, and nanoscale clustering patterns in individual presynaptic active zones. The variation in these properties affects the coupling of VGCCs with calcium sensors on SVs, synaptic efficacy, and temporal precision of transmission. In this study, we summarize how the morphological parameters of Ca_*V*_2 distribution obtained using SDS-FRL differ depending on the different types of synapses and could correspond to functional properties in synaptic transmission.

## Introduction

**FIGURE 1:**
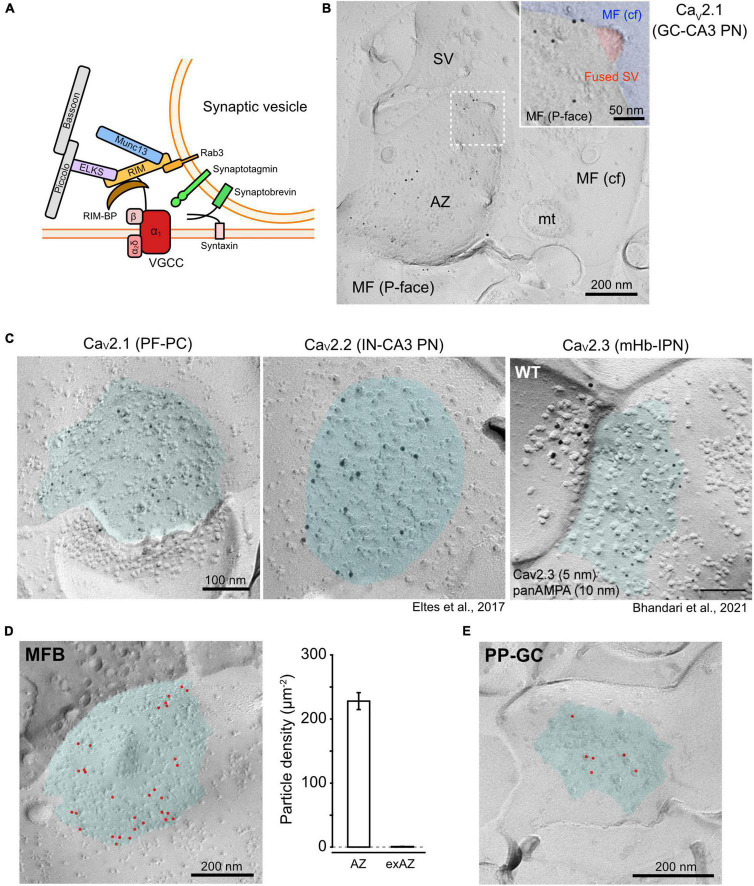
Distribution of Ca_*V*_2 channels at the presynaptic AZs. **(A)** A schema of a Ca_*V*_2 channel assembled with presynaptic proteins related to SV fusion. **(B)** An example EM image of Ca_*V*_2.1 particles concentrated in an AZ of a rat hippocampal dentate gyrus granule cell (GC)-CA3 pyramidal neuron (PN) synapse. Inset is a high-magnified image of the square area (dashed line) showing Ca_*V*_2.1 particles around a fused SV (indicated with red). **(C)** Distribution of Ca_*V*_2 subtypes in AZs of different synapse types. *Left*, labeling for Ca_*V*_2.1 at a PF-PC synapse in the mouse cerebellum. *Middle*, labeling for Ca_*V*_2.2 at an IN-CA3 PN synapse in the mouse hippocampus (Éltes et al., 2017). *Right*, labeling for Ca_*V*_2.3 (5 nm) and AMPA-type glutamate receptor (panAMPA, 15 nm) at an mHb-IPN synapse in the mouse brain (Bhandari et al., 2021). Cyan indicates AZ areas. **(D)** *Left*, Ca_*V*_2.1 labeling at the AZ in an MFB of P28 Wistar rat. Red points indicate gold particles. *Right*, a bar graph showing the density of Ca_*V*_2.1 particles in the AZs and non-AZ areas (non-AZs) of the rat MFBs. **(E)** Ca_*V*_2.1 labeling in a perforant path-GC synapse obtained from the same replica preparation as panel **(D)**. MF, mossy fiber; AZ, active zone; SV, synaptic vesicle; cf, cross-fracture; mt, mitochondria; PF, parallel fiber; PC, Purkinje cell; IN, interneuron; mHb, medial habenula; IPN, interpeduncular nucleus; MFB, MF bouton.

At a chemical synapse, the arrival of an action potential (AP) at the nerve terminal activates voltage-gated calcium channels (VGCCs), thereby inducing Ca^2+^ influx into the terminal. Consequently, the fusion of synaptic vesicles (SVs) into the plasma membrane is triggered and neurotransmitters are released. VGCCs are composed of a pore-forming α1 subunit that is encoded by 10 genes and classified into three subgroups characterized with electrophysiological and pharmacological properties: Ca_*V*_1 (L-type), Ca_*V*_2 (N, P/Q, and R-type), and Ca_*V*_3 (T-type) channels. The Ca_*V*_2 channels [Ca_*V*_2.1 (P/Q-type), Ca_*V*_2.2 (N-type), and Ca_*V*_2.3 (R-type)] are highly expressed in chemical synapses of the mammalian central nervous system (CNS) and are concentrated at the presynaptic active zone (AZ). The AZ consists of protein complexes of various molecules including target soluble N-ethylmaleimide-sensitive factor attached protein receptor (t-SNARE) proteins (syntaxin and SNAP-25) and scaffold proteins (e.g., RIMs, Munc-13, Bassoon, Piccolo, and ELKS) as well as VGCCs (Figure 1A). Fast and precise synaptic transmission is accomplished through the coupling of Ca_*V*_2 channels and the Ca^2+^-sensor proteins (e.g., synaptotagmins) of release-ready docked SVs, which are adjacent to each other (Figure 1B). The issue of the required number of Ca_*V*_2 channels for the fusion of a single SV is still under debate. Previous studies using squid giant synapses and chick ciliary ganglion calyx synapses have proposed that Ca^2+^ influx through a single VGCC can generate the fusion of an SV if the Ca^2+^ sensor is sufficiently close to the channel (Stanley, 2016). In chemical synapses of the mammalian CNS, however, Ca_*V*_2 channels make clusters in the AZs, indicating that the Ca^2+^ influx through multiple Ca_*V*_2 channels in a cluster generates the fusion of an SV.

In this study, we have highlighted recent progress toward understanding the distribution and functions of Ca_*V*_2 channels, mainly focusing on the findings based on an immunoelectron microscopic technique called sodium dodecyl sulfate-digested freeze-fracture replica labeling (SDS-FRL). SDS-FRL can be used to visualize the two-dimensional distribution of membrane molecules with high sensitivity and resolution (Masugi-Tokita et al., 2007) and is suitable for quantitative analysis of the number, density, and clustering of Ca_*V*_2 channels in the AZs. Among the three Ca_*V*_2 subunits, Ca_*V*_2.1 has been most extensively examined by several groups and, thus, is mainly discussed here. We have also described the technical notes of SDS-FRL for accurate cluster analysis and have discussed the functional roles of Ca_*V*_2 channel clustering on neurotransmitter release with different topographical models of Ca_*V*_2 channels and SVs.

## Observation of Ca_*V*_2 Channel Distribution in Active Zones Using Sodium Dodecyl Sulfate-Digested Freeze-Fracture Replica Labeling

Synaptic vesicles are thought to be docked near VGCCs in the AZs for the efficient triggering of their exocytosis in response to an AP. The geometric differences of Ca_*V*_2 channels, such as their clustering and the distance from docked SVs, characterize the variety of the presynaptic properties. However, due to the high-density protein accumulation in the AZs, the accessibility of antibodies against Ca_*V*_2 subtypes is potentially attenuated in the conventional immunofluorescence or pre-embedding immunoelectron microscopy. In contrast, the accessibility of the antibody to Ca_*V*_2 channels is improved in SDS-FRL because the epitopes are exposed on the two-dimensional fractured face of the membrane after most of the cytosolic proteins that could mask the epitopes are washed out during the SDS treatment. However, SDS-FRL relies on random fracturing of frozen slices, making the identification of labeled profiles more difficult compared with volume data obtained by the three-dimensional reconstruction of serial sections. SDS-FRL has been utilized for quantitative analysis of the nanoscale distribution of Ca_*V*_2 channels at the AZs in rodent brains (Holderith et al., 2012; Indriati et al., 2013; Baur et al., 2015; Nakamura et al., 2015; Éltes et al., 2017; Kusch et al., 2018; Luján et al., 2018a,b; Rebola et al., 2019; Eguchi et al., 2020; Kleindienst et al., 2020; Bhandari et al., 2021). Figure 1C shows the examples of the replica labeling for Ca_*V*_2.1, 2.2, and 2.3 on presynaptic boutons in different synapses (Éltes et al., 2017; Bhandari et al., 2021). The quantitative analysis of the gold particle distribution suggests that all subtypes of Ca_*V*_2 channels are accumulated in the AZs of presynaptic boutons. However, Cav2.3 has a high density in the surroundings of AZ too, which gradually decreases with distance from the AZ (Bhandari et al., 2021), whereas Cav2.1 shows very low density in the extra-AZ areas (Figure 1D).

## The Number and Density of Ca_*V*_2 Channels in Individual Active Zones

**FIGURE 2:**
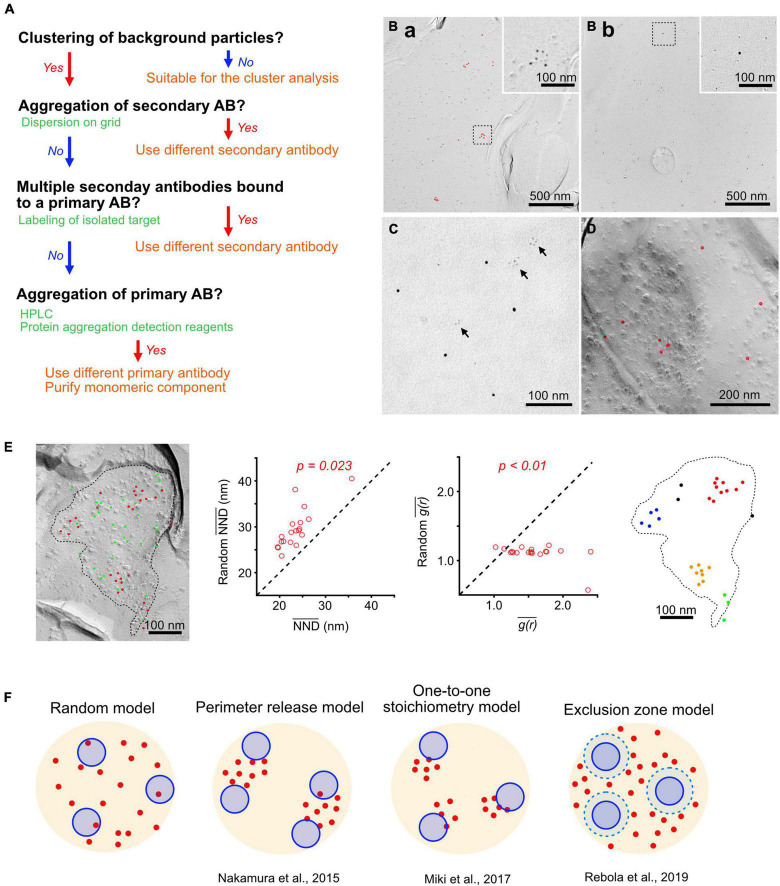
Cluster analysis of Ca_*V*_2 channels and the distribution models. **(A)** A flowchart for checking the artificial clustering of gold particles on replica samples. **(B)** Clustering of gold particles with two different anti-Ca_*V*_2.1 antibodies recognizing intracellular regions of Ca_*V*_2.1, which should give specific labeling on the protoplasmic face (P-face) of the replica. These particles represent non-specific background because they are found on the exoplasmic face (E-face) of the dendritic plasma membrane. Antibody A showed non-specific labeling with clusters of several gold particles **(Ba)**, whereas antibody B showed mostly isolated single background particles **(Bb)**. **(C)** Aggregation of secondary antibodies. The EM image of a mixture of goat anti-rabbit IgG conjugated with 2 nm gold particle and goat anti-guinea pig IgG conjugated with 5 nm gold particle dispersed on a grid coated with formvar. The secondary antibody with 2 nm gold particle had aggregations (arrows), whereas that with 5 nm gold particle did not. **(D)** Sparse distribution of gold particles labeling VGluT1 at the PF bouton of the mouse cerebellum, indicating binding of single gold particle-conjugated secondary antibody to a primary antibody. Red circles indicate the gold particles. **(E)** Cluster analysis of Ca_*V*_2.1. *Left*, an example of Ca_*V*_2.1 labeling at a PF-PC synapse of the mouse cerebellum. Red and green points indicate real and randomly simulated particles, respectively, in the AZ (dashed line). *Middle*, comparison of the mean NND (*left*) and *g(r)* values in each AZ obtained from the real and simulated particles. *P*-values were calculated by paired *t*-test. *Right*, cluster detection of the gold particles using DBSCAN with parameters of 3 for the minimum number of the particles in a cluster, and [t] mean NND + 2SD for the maximum distance (ε) allowed for a particle to be included in a cluster. Colors indicate different clusters except for black indicating non-clustered particles. **(F)** Schema showing AZ topography models for Ca_*V*_2 channels and release-ready docked SVs. *Random model*, random distribution of SVs and Ca_*V*_2 channels. *Perimeter release model*, multiple SVs are positioned at the perimeter of a Ca_*V*_2 channel cluster (Nakamura et al., 2015). *One-to-one stoichiometry model*, either 0 or 1 SV is docked per Ca_*V*_2 channel cluster (Miki et al., 2017). *Exclusion zone model*, Ca_*V*_2 channels are excluded from a zone around SVs (Rebola et al., 2019).

To estimate the absolute number of Ca_*V*_2 channels, a one-to-one relationship between single channels and gold particles in SDS-FRL would be ideal (labeling efficiency = 100%). However, the immunogold labeling efficiency in replica samples can be attenuated or amplified for various reasons. Underestimation could occur because not all channel proteins are allocated to the protoplasmic (P-) or exoplasmic (E-) faces for the detection by primary antibodies against the intracellular or extracellular epitopes, respectively. Even if all epitopes are present on the P- or E-faces, not all may be bound to primary antibodies because of masking by associated proteins or low avidity of the antibodies. The immunolabeling conditions including antibody concentrations, temperature of SDS treatment, and contents of blocking and antibody incubation solutions should be optimized too (Kaufmann et al., 2021). In contrast, overestimation could occur because of the aggregation of primary or secondary antibodies or multiple binding of these antibodies to a single target (Figure 2A). In our previous studies, we calibrated the labeling efficiency of SDS-FRL with a Ca_*V*_2.1 antibody in the cerebellum (Indriati et al., 2013; Miki et al., 2017) and calyx of Held (Nakamura et al., 2015), using the number of functional P/Q-type channels deduced by electrophysiological and Ca imaging data. However, some parameters used in the calibration were obtained in different species, ages, or brain regions. In addition, Ca^2+^ channels in the extra-AZ area were neglected. In this study, to estimate a more accurate labeling efficiency, we used Ca_*V*_2.1 labeling in mossy fiber boutons (MFBs) of dentate gyrus granule cells (GCs) innerving CA3 pyramidal neurons (PNs) in the rat hippocampus. Previous electrophysiological and ultrastructural studies have reported the total number of Ca_*V*_2.1 channels to be 1,320 per single MFB in rats at postnatal day (P) 21–22 (Li et al., 2007), and the total surface area of all AZs (∼30 AZs in an MFB) and extra-AZ as 3.33 and 75.5 μm^2^, respectively, in a single MFB of P28 rat (Rollenhagen et al., 2007). Using P28 rats, we labeled Ca_*V*_2.1 in MFBs using an anti-Ca_*V*_2.1 antibody (Frontier Institute, Supplementary Table 1 and Figure 1C), which is essentially the same as those used in our previous studies (Indriati et al., 2013; Nakamura et al., 2015; Luján et al., 2018a,b; Eguchi et al., 2020). The AZs in the MFBs were automatically demarcated using a deep learning-assisted software (Darea) with manual correction (Kleindienst et al., 2020) based on higher densities of intramembrane particles (IMPs) than extra-AZ on the P-face (Hagiwara et al., 2005; Figure 1D). The mean area of the complete AZs in the MFBs in replicas was ∼0.1 μm^2^, which was close to the values obtained by the three-dimensional reconstructions of the MFBs (0.09–0.13 μm^2^, Rollenhagen et al., 2007). The densities of gold particles labeling Ca_*V*_2.1 in the AZs and extra-AZs were 232 and 0.84 particles/μm^2^, respectively. This result and the area of AZ/extra-AZ suggest that 92% of Ca_*V*_2.1 channels are present in the AZs. Thus, the density of Ca_*V*_2.1 at AZs is estimated to be (1,320 channels × 0.92)/3.33 μm^2^ = 365 channels/μm^2^, and the Ca_*V*_2.1 labeling efficiency is calculated to be 64%. This value is very close to previously reported estimates using the same anti-Ca_*V*_2.1 antibody at rat cerebellar parallel fiber (PF)-Purkinje cell (PC) synapses (63%, Indriati et al., 2013; 62%, Miki et al., 2017) and at rat calyx of Held synapses (62%, Nakamura et al., 2015). Using the same Ca_*V*_2.1 antibody and similar labeling conditions, we compared the estimated number and density of Ca_*V*_2.1 channels in different types of CNS synapses (Supplementary Table 2). In the hippocampus, three types of excitatory synapses showed a range of 9.8–24.2 channels as per AZ but with similar densities (288–358 channels/μm^2^) and nearest neighbor distances (NND, an index for local density, 27–29 nm), indicating that the difference in number is mostly ascribable to that in the AZ size. Interestingly, the center-periphery index (CPI, Kleindienst et al., 2020) showed a significantly lower value (*P* < 0.0001) in perforant path-to-granule cell (PP-GC) synapses (0.42) than that in MFB synapses (0.67), indicating that Ca_*V*_2.1 is localized nearer to the AZ center in PP-GC synapses (Figures 1D,E). In the cerebellum, the Ca_*V*_2.1 density in PF boutons was higher in synapses made on molecular layer interneurons (MLI, 827 channels/μm^2^) than those on PCs (416 channels/μm^2^), indicating target cell-dependent regulation of Ca_*V*_2.1 density. Although the Cav2.1 numbers are linearly correlated with release probability in excitatory synapses of CA3 hippocampal pyramidal cells (Holderith et al., 2012), the number or density does not necessarily correlate with release probability or Ca^2+^ influx across different types of synapses (Éltes et al., 2017; Rebola et al., 2019). Functional properties of single channels or spatial relationship between Ca^2+^ channel clusters and release-ready docked SVs should also affect the synaptic efficacy and temporal precision of neurotransmission.

## Technical Consideration of Clustering Analysis Using Sodium Dodecyl Sulfate-Digested Freeze-Fracture Replica Labeling

Previous studies have demonstrated not only the number/density of Ca_*V*_2.1 channels in the AZs but also their clustering (Indriati et al., 2013; Nakamura et al., 2015; Miki et al., 2017; Rebola et al., 2019; Kleindienst et al., 2020), indicating the massive influx of Ca^2+^ through the clustered channels to effectively increase the Ca^2+^ level at the nano-spots in the AZs. To estimate clustering patterns accurately, a high labeling efficiency as described earlier is desirable. Moreover, some technical pitfalls causing artificial gold particle clustering must be excluded (Figure 2A). A valuable indicator of the artificial cluster formation is the non-specific background labeling of gold particles on the “wrong” face or cross-fracture (Figure 2B). In the case of our Ca_*V*_2.1 labeling with the antibody against an intracellular epitope, gold particles on the E-face could serve as background labeling. Figure 2B shows examples of such non-specific labeling with different anti-Ca_*V*_2.1 antibodies on the dendritic E-face in mouse cerebellar PCs. The gold particles with antibody A were clustered with 4–6 particles, whereas those with antibody B were mostly isolated single particles. If the background gold particles make clusters, the results of clustering analysis for the specific labeling could be misleading. Possible causes of this artificial clustering can be examined and eliminated as follows (Figure 2A).

### Aggregation of Antibodies

Immunoglobulin Gs (IgGs) are known to form aggregations due to various reasons, including acidic pH, often used for purification (Lopez et al., 2019). If the gold-conjugated secondary antibodies are aggregated, they make artificial clusters on the replica. The aggregation of secondary antibodies can be easily tested by observing the antibodies dispersed on a grid coated with formvar under electron microscope (EM) (Figure 2C). In this example, anti-rabbit IgG conjugated with 2 nm but not anti-guinea pig IgG conjugated with 5 nm gold particles formed clusters due to the antibody aggregation. This test also excludes the conjugation of multiple gold particles to a single secondary antibody. Checking the aggregation of primary antibodies is more time-consuming because it cannot be observed directly under EM unless they are negatively stained. Several analytical chemistry techniques such as high-performance liquid chromatography (HPLC) and fluorescent dyes for detecting aggregated proteins are suitable for detecting primary antibody aggregation (Liu et al., 2016; Oshinbolu et al., 2018). HPLC can be also used to purify the monomeric component of the primary antibody.

### Multiple Binding of Antibodies to a Single Protein

In immunofluorescence methods, the signal is enhanced when multiple secondary antibodies conjugated with fluorescent dyes bind to a single primary antibody. However, such a signal amplification hampers clustering analysis in SDS-FRL. To examine the possibility of this type of multiple particle binding to a single target, we labeled vesicular glutamate transporter 1 (VGluT1), which is an SV protein that is sparsely distributed on the PF bouton membrane in the mouse cerebellum (Miki et al., 2017). The 5 nm gold particles conjugated with anti-guinea pig IgG (the same one as we verified earlier) were sparsely distributed without clustering, indicating no multiple binding of the secondary antibody to a single primary antibody (Figure 2D). If all the possible reasons for artificial signal amplification described above are excluded, the last possibility is the multiple binding of primary antibodies to a single target protein. Although it should not often occur, since epitope regions in a single target protein are usually much smaller than the primary antibody, the use of monoclonal primary antibodies would help to avoid this problem.

## Cluster Analysis of Ca_*V*_2.1 Channels at Active Zones

A great variety of methods have been developed to detect particle clusters by comparison with random distributions; Szoboszlay et al. (2017) evaluated the benefits and effectiveness of the implemented methods, and they recommended the comparison of the mean NND between particles as a method to investigate whether gold particles form uniform or clustered patterns compared with random point patterns. If the particles are clustered, the mean NND will be significantly smaller than that of randomly distributed particles computed from Monte Carlo simulations. They also recommended the spatial autocorrelation function (ACF), described as *g(r)*, a derivative of Ripley’s *K* function based on all-to-all distances between particles, as another useful method to detect clustering. *g(r)* values close to 1 indicate the random distribution of particles, values < 1 indicate a uniform distribution, and values > 1 indicate clustering. In Figure 2E, we analyzed the Ca_*V*_2.1 clustering in the AZs at PF-PC synapses of the mouse cerebellum. We calculated the mean values of NND and *g(r)* of the real particles distributed within the AZs on the replicas and the simulated particles with random distribution (200 times simulations for each examined AZ). The mean values of real NND and *g(r)* were significantly smaller and larger, respectively, than their corresponding simulated values (*P* < 0.05, paired *t*-test, *n* = 20 AZs), suggesting that Ca_*V*_2.1 is distributed in a clustered manner within these AZs.

Several methods have been utilized to automatically detect each cluster of gold particles on the replica. A simple way to define a cluster is to consider particles that exist within a radius (e.g., 100 nm) of each particle to compose a cluster (Nakamura et al., 2015). An advanced method is density-based spatial clustering of applications with noise (DBSCAN), which is a density-based clustering algorithm that groups together points that are tightly clustered while marking points that are less dense and singly distributed as outliers (Szoboszlay et al., 2017). Figure 2E demonstrates the cluster analysis of Ca_*V*_2.1 immunolabeled AZs using DBSCAN with two parameters: the radius of a neighborhood (i.e., the maximum distance between two particles for one to be considered as in the neighborhood of the other) as mean NND plus two times its SD and the minimum number of particles necessary to form a cluster as 3. The DBSCAN algorithm detected 2.6 ± 0.2 clusters in PF-PC synapses (*n* = 65 AZs). The gold particle clusters consist of 6.8 ± 0.3 particles (*n* = 167 clusters), indicating that 10.6 ± 0.5 channels form a cluster in the AZs estimated with the labeling efficiency (64%).

Supplementary Table 2 shows the summary of the Ca_*V*_2.1 channel clustering, which was detected in all types of rodent CNS synapses analyzed so far in the previous and present studies using the same primary antibody (refer to the section “Materials and Methods” and Supplementary Table 1). Although the number and density of clusters vary depending on the synapse types, the mean NNDs and numbers of particles per cluster were quite similar, indicating a common mechanism of Ca_*V*_2.1 cluster formation. The distribution of Ca_*V*_2.1 clusters should be examined relative to docked SVs distribution for considering their functional implications (Nakamura et al., 2015). Several different models of the topographical relationship between Ca_*V*_2 channel clusters and docked SVs have been proposed as discussed in the following section (Figure 2F).

## Topographical Models of Ca_*V*_2 Clustering and Synaptic Vesicle Fusion

Voltage-gated calcium channels in the AZs are coupled with calcium sensors, synaptotagmins, on the docked SVs to efficiently evoke neurotransmitter release. Although it is still being debated about how many VGCCs are required to evoke a vesicle fusion, SDS-FRL observations have provided crucial information to understand the contribution of VGCC distribution to neurotransmitter release. At calyx of Held synapses in the rat auditory brainstem, the number of Ca_*V*_2.1 in clusters increased during hearing onset (P7 vs. P14) with no changes in NND, and the cluster area was expanded (Nakamura et al., 2015). This developmental change results in tighter coupling of Ca_*V*_2.1 channel clusters with vesicle fusion sites. A computational simulation based on this as well as electrophysiological and Ca^2+^ imaging results predicts that multiple docked SVs are located at the perimeter of a Ca_*V*_2.1 cluster, and the Ca_*V*_2.1 number in a cluster mainly determines the vesicular release probability (“perimeter release model,” Figure 2F). In contrast, at PF-MLI synapses of the mouse cerebellar cortex, NNDs between particles were significantly shortened during postnatal development (postnatal week 2 vs. 4) without changes of Ca_*V*_2.1 number in the cluster (Miki et al., 2017). This tighter VGCC arrangement may allow for more efficient coupling between Ca^2+^ entry and SV release. Interestingly, the numbers of SV docking sites (estimated from electrophysiological experiments) and Ca_*V*_2.1 clusters showed a close correspondence, leading to propose “one-to-one stoichiometry model” (Figure 2F). In contrast, Rebola et al. (2019) proposed that Ca_*V*_2.1 channels were not clustered but simply excluded from a 50 nm zone around docked SVs (“exclusion zone model,” Figure 2F) at cerebellar PF-PC synapses. However, this contrasts with observations in GABAergic stellate cell synapses, where SVs are tightly associated (∼10 nm) with the perimeter of VGCC clusters consistent with the perimeter release model (Rebola et al., 2019). In the exclusion zone model, the release probability is determined by the radius of the exclusion zones rather than the number of Ca_*V*_2.1 in the AZs.

## Conclusion

Sodium dodecyl sulfate-digested FRL studies demonstrate the nanoscale distribution of Ca_*V*_2 channels at AZs of presynaptic terminals. Although a single VGCC can generate the fusion of a single SV (Stanley, 2016), Ca_*V*_2 channels form clusters (approximately 10 channels) in the AZs of many synapse types in the CNS. The distribution of Ca_*V*_2 channels, especially topographical relationships between Ca_*V*_2 channel clusters and Ca^2+^ sensors of docked SVs, and the number of channels in a cluster modulate the properties of neurotransmitter release. The dynamic changes in the Ca_*V*_2 channel clustering might contribute to the presynaptic plasticity of synaptic transmission. It remains unclear how the distribution of Ca_*V*_2 channels and their topographical relationships with SVs are regulated in different types of synapses. The quantitative nanoscale analysis of Ca_*V*_2 channel distribution by SDS-FRL, combined with simultaneous visualization of the release-ready docked SVs, should be useful to unveil these questions.

## Data Availability Statement

The raw data supporting the conclusions of this article will be made available by the authors, without undue reservation.

## Ethics Statement

The animal study was reviewed and approved by the Ethics Committee of IST Austria.

## Author Contributions

KE and RS conceived and wrote the manuscript. All authors conducted electron microscopic experiments and contributed to the article and approved the submitted version.

## Conflict of Interest

The authors declare that the research was conducted in the absence of any commercial or financial relationships that could be construed as a potential conflict of interest.

## Publisher’s Note

All claims expressed in this article are solely those of the authors and do not necessarily represent those of their affiliated organizations, or those of the publisher, the editors and the reviewers. Any product that may be evaluated in this article, or claim that may be made by its manufacturer, is not guaranteed or endorsed by the publisher.
