# Supplementary material for: The Number and Distinct Clustering Patterns of Voltage-Gated Calcium Channels in Nerve Terminals
Source: Front Neuroanat. 2022 Feb 24;16:846615. doi: 10.3389/fnana.2022.846615 (PMC8907123; doi:10.3389/fnana.2022.846615)
Supplement: Supplementary file 1 [file Data_Sheet_1.PDF]

Supplementary materials

Materials and methods

Animals

Animal experiments were conducted in accordance with the guideline of the Institute of Science and Technology Austria (Animal license number: BMWFW-66.018/0012-WF/V/3b/2016). Mice were bred and maintained in the Preclinical Facility of IST Austria. C57BL/6J mice (postanatal (P) 5-7 weeks) and Wistar rats (P28 days) of either sex were used in this study.

High-pressure freezing of perfusion-fixed mouse brain slices

For perfusion-fixation, animals were anesthetized with ketamine/xylazine mixture via intraperitoneal injection and perfused phosphate-buffered saline (PBS) for 1 min followed by a fixative solution containing 2% PFA, 15% picric acid in 0.1 M sodium phosphate buffer (s-PB, pH 7.4) for 12 min at room temperature (23-25 °C). The brains were then removed from the skull and post-fixed for 0.5-1 h at RT. After washing the fixed brains with 0.1 M s-PB, sagittal slices of mouse cerebellum or coronal slices of rat forebrain including the hippocampus (140 µm thickness) were cut using a tissue slicer (Linear Slicer Pro 7, Dosaka, Kyoto, Japan) in 0.1 M s-PB at ice-cold temperature. The slices were washed with 0.1M s-PB and immersed in graded glycerol of 10-20% (for 10 min) and 30% (for 30 min) in 0.1M s-PB at RT for the cryoprotection. Small blocks of cerebellar lobule IV-VII or hippocampal dentate gyrus-CA3 region were trimmed from the fixed slices on silicone elastomer using a micro scalpel (#10316-14, FST), sandwiched with two copper carriers with a ring of double-sided tape (140-µm thickness), and then frozen by a high pressure freezing machine. The frozen samples were stored in liquid nitrogen until use.

SDS-digested freeze-fracture replica labeling (SDS-FRL)

The frozen brain tissues were fractured into two parts at -130 °C and replicated by carbon deposition (4-5 nm thick), carbon-platinum (uni-direction from 60°, 2 nm) and carbon (20-25

nm) in a freeze-fracture machine (JFD-V, JOEL, Tokyo, Japan). The samples were digested with 2.5% SDS solution containing 0.1 M Tris-HCl (pH 8.3) at 80°C for 18-22 h. The replicas were washed in the SDS solution and then a washing buffer (50 mM Tris-buffered saline (TBS, pH 7.4) containing 0.1% BSA) at RT. To avoid non-specific binding of antibodies, the replicas were blocked with 3% BSA, 2% cold fish skin gelatin (CFG) and 0.05% Tween-20 in TBS for 1 h at RT. The replicas were incubated with primary antibodies (Table S1) dissolved in a dilution buffer (1% BSA, 1% CFG, 0.05% Tween-20 in TBS) at 15°C 1-2 overnight, and then gold-nanoparticle conjugated secondary antibodies (Table S1) dissolved in the dilution buffer at 15°C overnight. After washing the replicas with the washing buffer, they were picked up onto a grid coated with formvar in distilled water. Images were obtained under TEM (Tecnai 10, FEI, Oregon, US) with RADIUS software (EMSIS GmbH, Münster, Germany).

Image analysis

Images were analyzed with Darea software (Kleindienst et al., 2020), Fiji, and R. The gold particle detection and AZ demarcation were performed on Darea software. AZs on P-face were indicated with the aggregation of intramembrane particles on the replica at electron microscopic level as described previously (Landis and Reese, 1974; Harris and Landis, 1986; Masugi-Tokita et al., 2007; Eguchi et al., 2020). The quantitative analysis of the particle distribution (e.g. density in AZs, nearest neighbor distance [NND], center-periphery index [CPI]) was performed using Darea. The particle cluster was defined by the DBSCAN method using the dbscan package of R (Hahsler et al., 2019). The minimum number of particles was set to 3 and the maximum distance between particles to the mean and 2 times the standard deviation of the NNDs in each AZ. Two-step Monte-Carlo simulation of randomly distributed particles (Kleindienst et al., 2020) and the calculation of *g(r)* was performed using the spatstat package of R (Baddeley et al., 2015).

Statistical analysis

All data were presented as mean ± standard error of means. Statistical analysis was performed with R (version 4.0.2, CRAN) on RStudio IDE (version 4.0.2). All p-values were

displayed in the figures.

Reference

Baddeley, A., Rubak, E., and Turner, R. (2015). *Spatial Point Patterns*. Philadelphia, PA: Chapman & Hall/CRC doi:10.1201/b19708.

Hahsler, M., Piekenbrock, M., and Doran, D. (2019). dbscan: Fast Density-Based Clustering with R. *J. Stat. Softw.* 91. doi:10.18637/jss.v091.i01.

Harris, K. M., and Landis, D. M. (1986). Membrane structure at synaptic junctions in area CA1 of the rat hippocampus. *Neuroscience* 19, 857–872.

Landis, D. M., and Reese, T. S. (1974). Differences in membrane structure between excitatory and inhibitory synapses in the cerebellar cortex. *J. Comp. Neurol.* 155, 93–125.

Table S1. Primary and secondary antibodies used in this study

**Antibody**	**Source**	**Catalog #**	**RRID**	**Conc.**	**Ref**
Gp anti-Ca_V2.1	Frontier Institute	MSFR106040	AB_2571851	4 or 8 µg/ml	Indriati et al., 2013; Nakamura et al., 2013; Miki et al., 2017; Eguchi et al., 2020
Gp anti-Ca_V2.1	Synaptic Systems	152 205	AB_2619842	2 µg/ml	Kleindienst et al., 2020
Gp anti-VGluT1	Frontier Institute	MSFR107440	AB_2571618	2 µg/ml	Miki et al., 2017
Gt anti-Gp IgG (5 nm GP)	BBI solutions	EMGAG5	N/A	1:30	
Gt anti-Rb IgG (2 nm GP)	BBI solutions	EMGAR2	N/A	1:30	

Gp: guinea pig, Rb: rabbit, Gt: goat, GP: gold particle

Table S2. $Ca_V2.1$ distribution in AZs of presynaptic terminals

				Ca_V2.1 channels					Ca_V2.1 cluster				
**Species**	**Region**	**Synapse**	**Age**	**N***	**Channel #†**	**Density† (μm^{-2})**	**NND (nm)**	**CPI**	**N****	**Cluster #**	**Density (μm^{-2})**	**Channel # in clusters†**	**Ref.**
Wistar rat	Hippocampus	MFB-CA3	28d	60	24.2	358.1	28.9	0.67	111	1.8	27.4	8.8	This study
		PP-GC	28d	44	9.8	288.3	27.9	0.42	39	0.9	22.5	9.2	
	Brainstem	Calyx of Held	7d	-	-	-	28	-	-	-	-	10.5	Nakamura et al., 2015
			14d	-	-	-	30	-	-	-	-	17.9	
C57BL/6J mouse	Hippocampus	CA3-CA1	14w	66	15.9	346.4	26.5	0.52	100	1.5	28.9	7.5	This study
	Cerebellum	PF-PC	28d	-	27.6	-	-	-	-	-	-	-	Indriati et al., 2013
			6w	65	32.2	415.5	28.9	0.57	167	2.6	33.2	10.6	This study
		PF-MLI	14-15d	-	27.1	579	19 (peak)	-	-	2.8	61.0	8.8	Miki et al., 2017‡
			29d	-	22.4	827	14 (peak)	-	-	2.4	85.5	9.1	

MFB: mossy fiber bouton, PP: perforant path, GC: dentate gyrus granule cell, PF: parallel fiber, PC: Purkinje cell, MLI: molecular layer interneuron

*Sample size (the number of synapses) of this study

**Sample size (the number of clusters) of this study

†The number and density of $Ca_V2.1$ were estimated by deviding those of immunogold particles for $Ca_V2.1$ with the labeling efficiency (0.62-0.64).

‡Minimum particle number for cluster detection was 2, whereas it was 3 for other studies

-: not available
